# A Synchronous Variable-Stroke Mechanism for Workspace Enhancement of a Four-Finger Soft Robotic Hand

**DOI:** 10.3390/biomimetics11050318

**Published:** 2026-05-03

**Authors:** Hui Chen, Zhenya Wang, Shikai Zhang, Ligang Yao

**Affiliations:** 1 Department of Mechanical Engineering, Faculty of Engineering, Universiti Malaya, Kuala Lumpur 50603, Malaysia; 2 Department of Mechanical Engineering, Tsinghua University, Beijing 100084, China; 3 School of Mechanical Engineering and Automation, Fuzhou University, Fuzhou 350108, China; ylgyao@fzu.edu.cn

**Keywords:** soft robotic hand, variable-stroke mechanism, pneumatic soft actuator, workspace analysis, grasping adaptability, multi-finger gripper

## Abstract

Soft robotic hands are well suited for handling fragile and geometrically diverse objects, yet many existing designs still rely on fixed finger layouts, which limits grasping adaptability when object size varies substantially. To address this issue, this study proposes a four-finger pneumatic soft robotic hand with a synchronous variable-stroke base mechanism. The design combines a rigid reconfigurable base with compliant soft fingers, allowing the radial positions of the fingers to be adjusted before grasping. A system-level kinematic model is established to describe the relationship between base stroke, finger bending, and the reachable workspace of the hand. A prototype is fabricated, and comparative grasping experiments are conducted under fixed-stroke and variable-stroke configurations using objects with different grasping cross-sections. The results show that the proposed mechanism achieves stable geometric reconfiguration and improves grasping performance when the initial finger spacing is matched to the object size. In particular, the variable-stroke configuration provides better grasp stability and a wider usable grasping range than the fixed-stroke configuration. These findings indicate that geometric reconfiguration at the hand level is an effective way to enhance the adaptability of multi-finger soft robotic hands.

## 1. Introduction

As robotic manipulation extends from structured production lines to less predictable working conditions, end-effectors are increasingly required to handle objects that differ in size, shape, stiffness, and surface condition. Under these circumstances, rigid grippers often perform well only when the target geometry is known in advance and the contact condition remains well controlled. Their limitation becomes more apparent when the object is fragile, deformable, or geometrically irregular. Soft robotic hands address this issue by using compliant materials to generate motion and contact, which allows safer interaction and more tolerant surface adaptation during grasping [1,2,3,4,5,6,7,8].

A substantial body of research has improved pneumatic soft manipulators at the actuator and finger levels [2]. Mosadegh et al. introduced PneuNet actuators for rapid chamber-driven bending [9]. Galloway et al. showed that embedded fiber reinforcement can be used to program bending radius and motion direction [10]. Polygerinos et al. established analytical and finite-element models for fiber-reinforced soft bending actuators, which provided a clearer link between geometry, pressure input, and mechanical output [11]. Tawk et al. further integrated sensing and control into a monolithic soft finger, extending soft-finger research beyond actuation alone [12]. Taken together, these studies advanced structural design, deformation modeling, and sensing integration. Their common focus, however, remained the actuator or the individual finger. In most cases, the global layout of the hand was still treated as fixed. As a result, the overall grasping envelope of the hand was largely determined by a preset finger distribution, which restricts adaptability when target objects vary substantially in grasping cross-section [13,14,15].

Beyond actuator-level improvements, grasping adaptability has also been addressed through underactuated hands, variable-stiffness structures, compliant gripper surfaces, and reconfigurable finger layouts [16]. These approaches have substantially improved the ability of robotic grippers to interact with objects of different shapes. However, they usually enhance adaptability in different ways. Compliance-based and underactuated grippers mainly rely on passive deformation or joint coupling after contact has occurred [17]. Variable-stiffness grippers regulate contact behavior by changing structural rigidity [18]. Reconfigurable rigid or hybrid hands can adjust grasping geometry more directly, but they often require additional joints, multiple actuators, independent finger-positioning modules, or more complex control and transmission structures [19]. Therefore, a gap remains for a compact mechanism that can reconfigure the global finger layout of a multi-finger soft hand before grasping, while preserving the same soft-finger modules.

This limitation is central to the present study. A soft finger with favorable bending behavior does not by itself guarantee that a multi-finger hand can approach, enclose, and retain objects of different sizes under equally suitable initial conditions. When the initial spacing among fingers is fixed, the hand must rely on finger deformation alone to compensate for geometric mismatch. That strategy is often sufficient for moderate cases, but it becomes less reliable when the object is either markedly smaller or noticeably larger than the nominal grasping geometry. In that situation, the missing element is not another actuator-level optimization, but a mechanism that can adjust the hand-level geometry before contact is established. This is the specific gap addressed here. The problem is therefore different from our previous work, which focused on the wave-shaped contour actuator itself, including actuator design, modeling, simulation, and grasp verification at the finger level [20].

Against this background, the present work investigates a four-finger soft robotic hand with a synchronous variable-stroke base mechanism. The central idea is to improve grasp adaptability by reconfiguring the initial geometry of the hand, rather than by further modifying the soft finger itself. The study makes three contributions. First, it proposes a compact synchronous radial transmission mechanism that converts the output of a single swing-cylinder-driven input into coordinated radial motion of four finger roots. This mechanism allows the finger spacing to be adjusted before grasping while keeping the pneumatic soft fingers unchanged. Second, it establishes a system-level workspace model that relates base-stroke variation, finger bending, and the reachable region of the hand. Third, it validates the proposed design through comparative experiments under fixed-stroke and variable-stroke configurations. The novelty therefore lies in hand-level geometric reconfiguration through a shared synchronous transmission, rather than in actuator-level optimization alone.

The remainder of this paper is organized as follows. Section 2 presents the design of the synchronous variable-stroke mechanism. Section 3 establishes the workspace model and analyzes the effect of stroke variation on grasping range. Section 4 describes the prototype fabrication and experimental setup. Section 5 reports the comparative grasping results and discussion. Finally, Section 6 concludes the paper.

## 2. System Design of the Variable-Stroke Soft Robotic Hand

### 2.1. Overall Architecture

**Figure 1:**
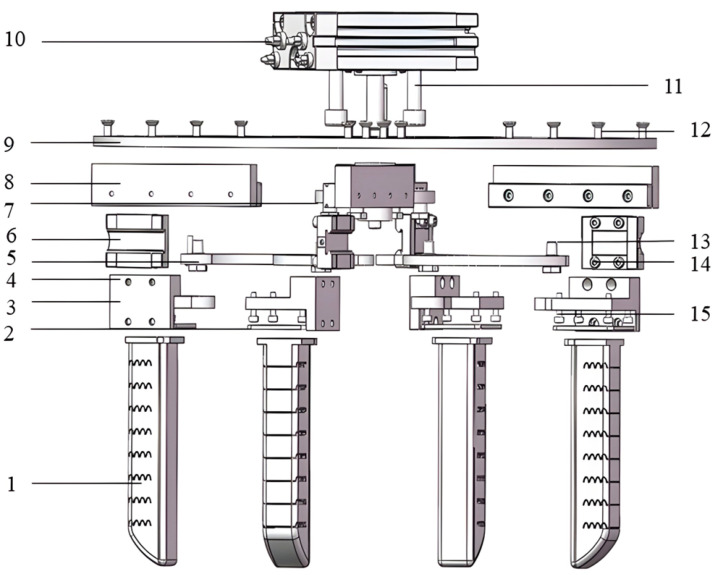
Exploded view of the proposed variable-stroke soft robotic hand: 1, pneumatic soft finger actuator; 2, actuator mounting clamp plate; 3, actuator mounting plate; 4, M3 threaded hole; 5, arc-shaped movable link; 6, actuator and guide-rail fixing plate; 7, shaft flange; 8, guide-rail mounting vertical plate; 9, main mounting base plate; 10, thin swing cylinder; 11, M8 bolt; 12, M4 bolt; 13, M6 bolt; 14, M3 threaded hole; 15, M3 bolt.

The proposed robotic hand consists of a compliant four-finger grasping module and a synchronous variable-stroke base mechanism. The four soft fingers are symmetrically distributed around the center of the base and jointly form a compact multi-finger grasping system. In this configuration, the soft fingers provide compliant bending and conformal contact with target objects, whereas the base mechanism is responsible for coordinated adjustment of the finger positions before grasping. As a result, the system combines local compliance at the finger level with global geometric adaptability at the hand level. The overall exploded structure of the proposed hand is shown in Figure 1.

The soft fingers used in this hand are chamber-based pneumatic bending actuators. Each finger consists of an upper expandable chamber layer and a lower constraint layer. When compressed air is supplied to the internal chambers, the expandable layer undergoes larger deformation than the constraint layer. This deformation mismatch generates bending toward the constraint side. As a result, the finger can form compliant contact and partial enclosure around the target object without using rigid joints or tendon-driven phalanges.

The hand is designed to address a limitation commonly observed in fixed-base soft robotic grippers. In a conventional fixed-spacing configuration, the achievable grasping range is constrained by the initial arrangement of the fingers, even if each individual finger exhibits favorable bending performance. In contrast, the present design introduces a variable-stroke base such that the relative positions of the four fingers can be synchronously adjusted according to the size of the target object. The design objective of this architecture is to increase the effective grasping range of the hand without changing the soft-finger structure. This is achieved by separating the global positioning function from the local bending function: the rigid base adjusts the initial radial spacing of the four fingers, while the pneumatic soft fingers provide compliant deformation and surface adaptation during grasping.

For a symmetric four-finger arrangement, the angular position of the i-th finger root can be defined as

(1) $$\begin{array}{cccc} & \alpha_{i}=(i-1)\frac{\pi }{2},i=1,2,3,4 & & \end{array}$$

This definition reflects the uniform angular distribution of the four fingers around the center of the base and provides the basic geometric reference for the subsequent kinematic model. And the planar position of the i-th finger base is written as

(2) $$\begin{array}{cccc} & p_{i}^{\;b}\left(s\right)=\left[\begin{array}{c} \left(r_{0}+s\right)\cos\alpha_{i}\\ \left(r_{0}+s\right)\sin\alpha_{i}\\ 0 \end{array}\right],s\in \left[s_{min},s_{max}\right] & & \end{array}$$

where $r_{0}$ denotes the nominal radial distance from the center of the base to the finger root, and s is the stroke adjustment imposed by the synchronous base mechanism. In this study, the adjustable stroke range satisfies

(3) $$\begin{array}{cccc} & \Delta s=s_{max}-s_{min}=80\;\mathrm{mm} & & \end{array}$$

which corresponds to the radial adjustment range between the nearest-stroke and farthest-stroke configurations of the proposed base.

### 2.2. Synchronous Variable-Stroke Base Mechanism

**Figure 2:**
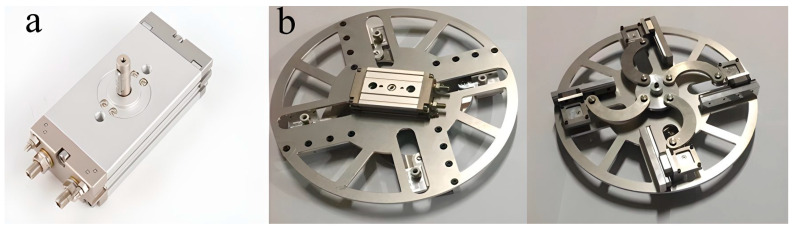
Structural components of the synchronous variable-stroke base: (**a**) thin swing cylinder; (**b**) top and bottom views of the circular base plate.

The variable-stroke base is designed as a rigid aluminum-alloy structure to support the upper pneumatic actuation module and to ensure stable transmission during repeated motion. A thin swing cylinder (CDRQ2BS-20) is mounted on the upper side of the circular base plate and serves as the driving source of the stroke-adjustment mechanism. The cylinder output is transmitted to a central shaft flange, which drives four arc-shaped movable links. Each link is connected to one finger fixture and constrains the corresponding finger module to move along a radial guide rail. As a result, the four finger roots can be repositioned synchronously while remaining evenly distributed around the center of the hand. This actuator is compact, structurally integrated, and suitable for long-term operation with reliable rotational precision. The corresponding components are shown in Figure 2a. The main carrier of the mechanism is a circular aluminum-alloy base plate, as shown in Figure 2b. The circular layout is adopted to match the symmetric arrangement of the four fingers. Four guide rails are distributed on the base plate so that each finger module can move along a prescribed radial direction. Each rail is associated with threaded holes for mounting the guide-rail support and the finger fixture. To reduce mass without sacrificing structural integrity, eight sector-shaped hollow regions are introduced into the base plate. The circular base plate is fabricated from aluminum alloy to provide sufficient stiffness for supporting the upper swing cylinder and the transmission components. Four radial guide rails are arranged on the plate to guide the motion of the finger modules. To reduce structural mass while preserving the load-bearing layout, eight sector-shaped hollow regions are introduced into the plate. This hollowed design reduces the plate mass by approximately 35% compared with the non-hollowed configuration. The nearest and farthest radial positions of the finger roots are 65 mm and 145 mm, respectively, giving an adjustable radial stroke of 80 mm. A shaft flange is arranged beneath the plate and serves as the central transmission interface between the swing cylinder and the four arc-shaped movable links. These movable links are further connected to the individual finger fixtures, thereby enabling coordinated stroke variation in all four fingers.

**Figure 3:**
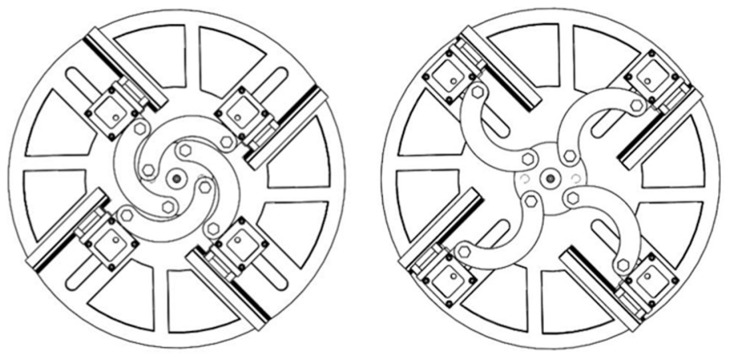
Minimum-stroke and maximum-stroke configurations of the proposed hand.

The working principle of the mechanism is illustrated in Figure 3. When pneumatic pressure is supplied to the thin swing cylinder, the cylinder output drives the shaft flange, which in turn transmits motion to the four arc-shaped movable links. Under the constraint of the guide rails, the finger modules move synchronously between the minimum-stroke and maximum-stroke configurations. In this way, the relative spacing among the four fingers can be adjusted before grasping, allowing the hand to accommodate objects with different characteristic sizes. The overall mechanism enables repeated radial repositioning of the four finger modules within an adjustable stroke range of 80 mm, corresponding to the transition between the nearest-stroke and farthest-stroke configurations.

From a mechanism viewpoint, the motion transmission from the cylinder to the finger base can be represented in a compact form as

(4) $$\begin{array}{cccc} & s=g\left(q\right) & & \end{array}$$

where q denotes the output displacement of the swing-cylinder-driven transmission chain and g(⋅) is the stroke mapping determined by the shaft flange, arc-shaped movable links, and guide-rail constraints. Since the primary purpose of this section is structural description, the exact closed-form expression of g(⋅) is not expanded here; instead, the mechanism is characterized by its monotonic and bounded stroke-adjustment behavior within the operating range defined in Equation (3).

A key advantage of this design is the separation of functions between the soft fingers and the rigid base. The soft fingers remain responsible for compliant bending and local surface adaptation, while the synchronous variable-stroke base regulates the global finger spacing. This functional decomposition improves modularity and allows the hand to handle both relatively small and relatively large objects more effectively than a fixed-base configuration. The aluminum-alloy base was selected because the base must simultaneously support the upper swing cylinder, constrain the four guide rails, and maintain transmission accuracy during repeated stroke adjustment. This rigid support is important because geometric reconfiguration occurs before grasping, and any structural compliance or asynchronous motion at the base would directly affect the initial finger spacing.

### 2.3. Geometric Description of the Variable-Stroke Configuration

To provide a concise kinematic interface for the workspace analysis in the next section, the fingertip position can be expressed as the superposition of base motion and finger bending. Let $\theta_{i}$ denote the bending angle of the i-th soft finger and let $l_{e}$ denote its effective bending length. Then the fingertip position of the i-th finger can be written as

(5) $$\begin{array}{cccc} & p_{i}^{\;t}\left(s,\theta_{i}\right)=p_{i}^{\;b}\left(s\right)+R_{z}\left(\alpha_{i}\right)\left[\begin{array}{c} l_{e}\sin\theta_{i}\\ 0\\ l_{e}\left(1-\cos\theta_{i}\right) \end{array}\right] & & \end{array}$$

where $R_{z}(\alpha_{i})$ is the planar rotation matrix associated with the finger orientation angle $\alpha_{i}$. Equation (5) indicates that the reachable space of each fingertip depends on both the variable stroke s and the finger bending angle $\theta_{i}$. Therefore, unlike a fixed-base design, the global grasping workspace of the proposed hand is jointly determined by the rigid-base reconfiguration and the compliant bending deformation of the fingers. This mechanism–deformation coupling is the basis of the workspace analysis developed in the next section. This formulation provides a system-level kinematic interface between the reconfigurable base and the soft-finger deformation. The base stroke determines the initial root position of each finger, whereas the bending angle determines the local fingertip displacement. Therefore, the overall reachable region is obtained by combining radial base adjustment with constant-curvature finger bending.

For two opposite fingers, the effective grasping span can be approximated as

(6) $$\begin{array}{cccc} & D_{opp}\left(s\right)=2\left(r_{0}+s\right) & & \end{array}$$

which shows that increasing the stroke directly enlarges the nominal object-accommodation range of the hand. Similarly, the distance between two adjacent finger roots is which shows that changing the stroke directly tunes the nominal object-accommodation range of the hand.

(7) $$\begin{array}{cccc} & D_{adj}\left(s\right)=\sqrt{2}\left(r_{0}+s\right) & & \end{array}$$

and therefore also increases monotonically with the stroke. This monotonic relation describes the nominal geometry of the hand, rather than a monotonic improvement in grasp performance. Although Equations (6) and (7) are simplified geometric relations, they clearly reflect the design intent of the proposed mechanism, namely, to expand the global workspace through synchronous finger-base adjustment rather than through actuator redesign alone.

Overall, the proposed variable-stroke hand achieves system-level geometric reconfiguration while retaining the compliant characteristics of pneumatic soft fingers. This combination provides the mechanical foundation for subsequent workspace evaluation and comparative grasping experiments.

## 3. Workspace Modeling and Analysis

### 3.1. Kinematic Modeling of the Variable-Stroke Hand

**Figure 4:**
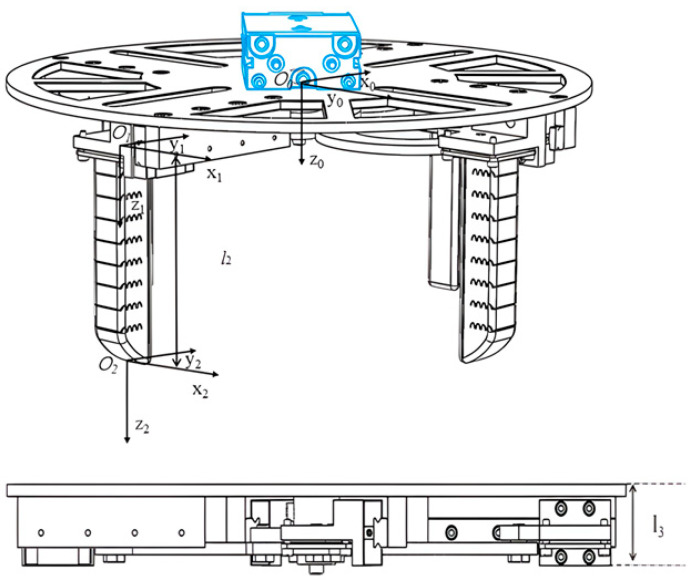
Coordinate definition and geometric parameters of the proposed hand.

To analyze the reachable region of the proposed hand, a compact kinematic model is established at the system level. The base coordinate frame is defined with its origin at the center of the circular base plate. The positive x-axis is aligned with the centerline of one radial guide rail and points outward from the base center. The positive z-axis points vertically downward, and the y-axis is determined according to the right-hand rule. The positive x-axis is defined along the centerline of the guide rail and points away from the base center, the positive z-axis points vertically downward, and the y-axis is determined by the right-hand rule. The corresponding coordinate-frame definition and principal geometric parameters are illustrated in Figure 4. In the present design, the vertical offset between the base frame and the finger-root frame is *l*_3_ = 27 mm, and the effective finger length is *l*_2_ = 115 mm.

**Figure 5:**
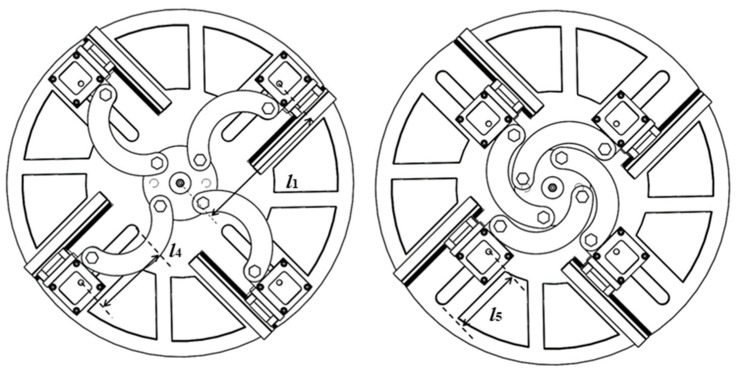
Minimum-stroke and maximum-stroke configurations of the variable-stroke base.

The radial position of the finger root is controlled by the synchronous variable-stroke base. According to the geometric definition shown in Figure 5, the nearest-stroke and farthest-stroke radii are *l*_5_ = 65 mm and *l*_1_ = 145 mm, respectively, yielding a total stroke range of

(8) $$l_{4}=l_{1}-l_{5}=80\;\mathrm{mm}$$

Let $s\in [0,l_{4}]$ denote the stroke variable measured from the nearest-stroke configuration. Then the radial distance from the base center to the root of each finger can be written as

(9) $$\begin{array}{cccc} & \rho (s)=l_{5}+s & & \end{array}$$

Accordingly, the position vector of the i-th finger root in the base frame is

(10) $$\begin{array}{cccc} & p_{b_{i}}\left(s\right)=\left[\begin{array}{c} \rho \left(s\right)\cos\alpha_{i}\\ \rho \left(s\right)\sin\alpha_{i}\\ l_{3} \end{array}\right] & & \end{array}$$

Equation (10) describes the system-level reconfiguration introduced by the variable-stroke base. Unlike a fixed-base design, the present hand allows the finger roots to move radially before the bending deformation of the soft fingers is activated. As shown in Figure 5.

At the finger level, the soft actuator is modeled under the constant-curvature assumption. Let κ denote the curvature of a finger and let θ denote the corresponding bending angle. Their relationship is

(11) $$\begin{array}{cccc} & \theta =\kappa l_{2} & & \end{array}$$

and the corresponding bending radius is

(12) $$\begin{array}{cccc} & R=\frac{1}{\kappa } & & \end{array}$$

For a planar constant-curvature segment, the fingertip position in the local finger frame is expressed as:

(13) $$\begin{array}{cccc} & p_{t}^{\;loc}\left(\theta \right)=\left[\begin{array}{c} R\left(1-\cos\theta \right)\\ 0\\ \mathrm{Rsin}\theta \end{array}\right] & & \end{array}$$

The global fingertip position of the i-th finger is then given by

(14) $$\begin{array}{cccc} & p_{t_{i}}\left(s,\theta \right)=p_{b_{i}}\left(s\right)+R_{z}\left(\alpha_{i}\right)\;p_{t}^{\;loc}\left(\theta \right) & & \end{array}$$

where $R_{z}(\alpha_{i})$ is the rotation matrix about the z-axis, namely,

(15) $$\begin{array}{cccc} & R_{z}\left(\alpha_{i}\right)=\left[\begin{array}{ccc} \cos\alpha_{i} & -\sin\alpha_{i} & 0\\ \sin\alpha_{i} & \cos\alpha_{i} & 0\\ 0 & 0 & 1 \end{array}\right] & & \end{array}$$

Equation (15) shows that the fingertip trajectory is jointly determined by the base stroke s and the finger bending angle θ. This coupling between rigid-base reconfiguration and compliant finger bending is the main kinematic feature of the proposed hand.

For geometric interpretation, the nominal distance between two opposite finger roots is

(16) $$\begin{array}{cccc} & D_{opp}\left(s\right)=2\rho \left(s\right) & & \end{array}$$

and the nominal distance between two adjacent finger roots is

(17) $$\begin{array}{cccc} & D_{adj}(s)=\sqrt{2}\;\rho (s) & & \end{array}$$

These expressions indicate that the global geometric envelope of the hand increases monotonically with the stroke. Therefore, the variable-stroke mechanism enlarges the nominal grasping range even before the fingers start bending.

### 3.2. Workspace Computation and Interpretation

Based on the above model, the reachable workspace of the proposed hand is defined as the union of all admissible fingertip positions generated by the stroke variable s and the bending angle θ, namely,

(18) $$\begin{array}{cccc} & W=\overset{4}{\underset{i=1}{⋃}}\left\{p_{t_{i}}(s,\theta )\;\;|\;\;0\leq s\leq l_{4},\;\;\theta_{min}\leq \theta \leq \theta_{max}\right\} & & \end{array}$$

In practice, the workspace is obtained by discretizing the stroke and bending angle within their admissible ranges and evaluating Equation (18) point by point. This procedure yields the reachable region of a single finger and, subsequently, the overall workspace of the four-finger hand.

**Figure 6:**
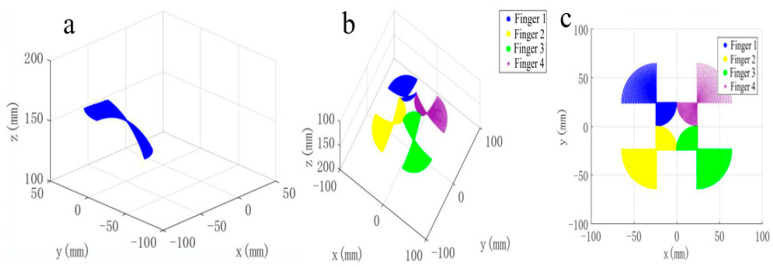
Computed workspace of the proposed hand: (**a**) single-finger reachable region; (**b**) 3D four-finger workspace; (**c**) 2D four-finger workspace.

The computed workspace reveals two key features. First, the synchronous variable-stroke base expands the radial distribution of the finger roots from 65 mm to 145 mm, thereby enlarging the global geometric envelope of the hand. Second, the bending deformation of each soft finger further extends the reachable region from the root positions into a continuous grasping domain. As a result, the final workspace is determined by the coupling of rigid-base reconfiguration and compliant finger bending, rather than by finger bending alone, as shown in Figure 6.

The workspace result also indicates that the influence of the bending angle on the reachable region is not strictly monotonic. As the bending angle increases, the workspace first expands and then contracts due to geometric convergence of the fingers. This trend reflects the balance between fingertip extension and inward enclosure during bending.

Under the selected structural parameters, the overall workspace indicates that the proposed four-finger hand can accommodate target objects with characteristic size below 116 mm. Moreover, because the hand can switch continuously between nearest-stroke and farthest-stroke configurations, it is able to adapt to objects with substantially different dimensions. This result provides the geometric basis for the comparative grasping experiments presented in the following sections. It should be noted, however, that the computed workspace describes geometric accessibility rather than grasp stability itself. Actual grasp performance also depends on contact distribution, enclosure quality, friction condition, and the force generated by the soft fingers during lifting.

## 4. Experimental Setup

### 4.1. Prototype Fabrication and Assembly

A prototype of the proposed four-finger soft robotic hand was fabricated to validate the variable-stroke design and its grasping capability. The overall system consists of a rigid variable-stroke base and four pneumatic soft fingers. The base was manufactured from aluminum alloy to ensure sufficient stiffness during stroke adjustment, since the thin swing cylinder was mounted on the upper part of the mechanism and the base had to support both the actuator weight and the transmission load. To achieve the required dimensional accuracy and assembly quality, the base components were fabricated by CNC milling according to the two-dimensional drawings derived from the SolidWorks model.

The soft fingers were fabricated separately and then assembled onto the base through the flange interface. In the present manuscript, the detailed molding procedure of the single-finger actuator is not repeated, since the actuator fabrication process has been reported in the previous study. Here, only the assembled hand is considered as the experimental object. After assembly, the four fingers were symmetrically distributed around the center of the base and connected to the synchronous variable-stroke mechanism through the corresponding fixtures and movable links.

The stroke-adjustment function of the prototype was realized by the thin swing cylinder mounted on the top of the base. By driving the shaft flange and the arc-shaped movable links, the mechanism could synchronously reposition the four finger roots between the nearest-stroke and farthest-stroke configurations.

### 4.2. Experimental Platform and Test Protocol

The experimental platform was established to evaluate the practical grasping performance of the proposed hand under different object sizes and shapes. During the tests, the four-finger soft robotic hand was fixed in place, and pneumatic input was supplied to the soft fingers through the corresponding air circuit. The grasping experiments focused on everyday objects with distinct geometric features, including spherical, cylindrical, box-shaped, flexible, and irregular objects. The tested objects and their basic physical properties are summarized in Table 1.

The experimental validation was organized in two parts. The first part aimed to verify the stroke-adjustment function of the mechanism. In this part, the hand was actuated to switch between the nearest-stroke and farthest-stroke states, and the coordinated motion of the four fingers was observed. This test was used to confirm that the synchronous variable-stroke base operated as intended and that the finger positions could be adjusted before grasping.

The second part consisted of grasping experiments with different objects. For each trial, the target object was placed within the central region of the hand, and the stroke configuration was selected according to the object size. Pneumatic input was then applied to the four fingers to perform enveloping grasping. During the tests, particular attention was paid to whether the hand could achieve stable contact with the target object and maintain the grasp without visible slip or drop. By adjusting the position of the arc-shaped movable link, the hand could perform variable-stroke grasping for objects with substantially different dimensions. This procedure was adopted to evaluate the practical benefit of the proposed variable-stroke configuration.

The selected objects covered spherical, cylindrical, cubic, deformable, and box-shaped targets, which were sufficient to assess the adaptability of the proposed hand under non-uniform grasping conditions.

### 4.3. Evaluation Metrics

To evaluate the performance of the proposed hand, three metrics were considered: grasp success rate, stable holding rate, and size adaptability.

The grasp success rate is defined as

(19) $$\begin{array}{cccc} & \eta_{g}=\frac{N_{g}}{N_{t}}\times 100\% & & \end{array}$$

where $N_{g}$ is the number of successful grasping trials and $N_{t}$ is the total number of trials.

The stable holding rate is defined as

(20) $$\begin{array}{cccc} & \eta_{s}=\frac{N_{s}}{N_{t}}\times 100\% & & \end{array}$$

where $N_{s}$ is the number of trials in which the object could be stably held after lifting without visible slip or drop during the observation interval.

To characterize system-level adaptability, the size adaptability is evaluated by the covered characteristic-size range of the objects that can be stably grasped under a given configuration, namely

(21) $$\begin{array}{cccc} & \Delta L=L_{max}-L_{min} & & \end{array}$$

where $L_{max}$ and $L_{min}$ denote the maximum and minimum characteristic sizes of the objects that can be stably grasped, respectively. In this study, the characteristic size $L_{c}$ of an object is defined as the maximum dimension in the grasping cross-section rather than the global maximum outer dimension.

For the geometric verification in Section 5.1, the percentage error between the theoretical span and the measured span is calculated as

(22) $$\begin{array}{cccc} & \varepsilon =\frac{|x_{meas}-x_{theory}|}{x_{theory}}\times 100\% & & \end{array}$$

where x denotes either the opposite-finger span $D_{opp}$ or the adjacent-finger span $D_{adj}$.

These metrics were selected because they directly reflect the objective of the present work, namely, to improve the grasping range and adaptability of the hand through synchronous variable-stroke reconfiguration.

## 5. Results and Discussion

### 5.1. Geometric Verification of the Synchronous Variable-Stroke Mechanism

**Figure 7:**
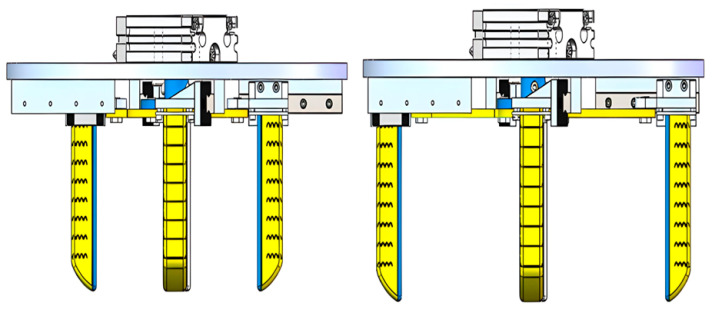
Prototype of the proposed four-finger soft robotic hand under different stroke configurations.

The first set of experiments was conducted to verify the geometric reconfiguration capability of the synchronous variable-stroke base. As shown in Figure 7, the prototype could be adjusted stably between the nearest-stroke and farthest-stroke configurations, and the four finger roots moved synchronously during the stroke transition. No obvious asynchronous motion, structural interference, or transmission instability was observed during the adjustment process.

To quantify this behavior, the theoretical and measured finger-root spans under three representative stroke settings are compared in Table 2. The measured opposite-finger span and adjacent-finger span followed the same monotonic variation trend as theoretical predictions. The deviations between theoretical and measured values remained limited over the tested stroke range, indicating that the proposed transmission mechanism can achieve the intended geometric reconfiguration with acceptable accuracy.

This result is important for the subsequent grasping analysis. The present design does not rely solely on finger deformation to adapt to target objects. Instead, it first changes the initial geometric distribution of the fingers through base-stroke adjustment and then uses compliant bending to establish contact. Therefore, the experimental verification in Table 2 provides direct support for the workspace model developed in Section 3 and establishes the mechanical basis for the grasping experiments discussed next.

### 5.2. Comparative Grasping Performance Under Fixed-Stroke and Variable-Stroke Configurations

**Figure 8:**
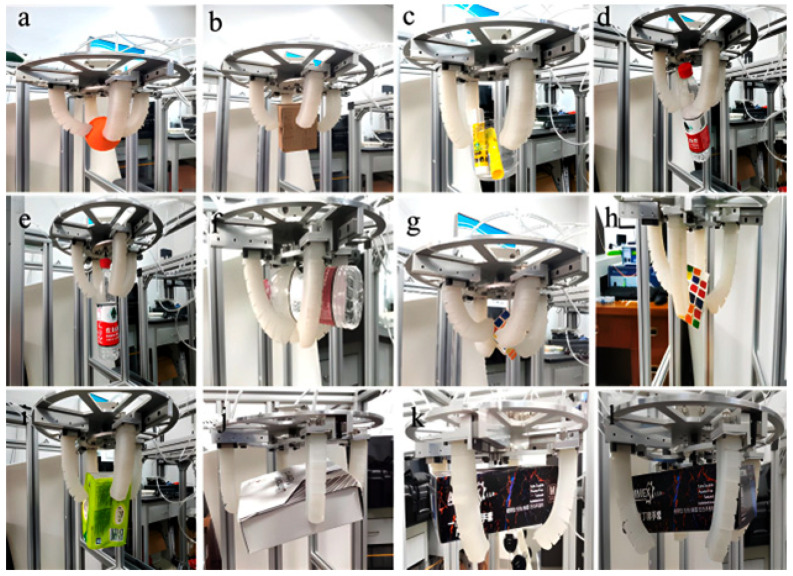
Representative grasping results for the tested objects with different sizes, shapes, and grasping configurations: (**a**) orange; (**b**) rectangular box A; (**c**) beverage can; (**d**–**f**) mineral water bottle under different grasping configurations; (**g**,**h**) Rubik’s cube under two grasping configurations; (**i**) tissue pack; (**j**) rectangular box B; and (**k**,**l**) rectangular box C under two grasping configurations.

The grasping performance of the proposed hand under fixed-stroke and variable-stroke configurations is summarized in Table 3, and representative results are shown in Figure 8. For consistency, the fixed-stroke configuration was kept at $s_{f}=40\;\mathrm{mm}$ for all objects, whereas the variable-stroke configuration was adjusted according to the object size. The comparison focuses on grasp success, holding stability, and the ability of the hand to accommodate targets with different grasping cross-sections.

The results show a clear difference between the two configurations. Under the fixed-stroke setting, the hand could grasp several objects of moderate size, but its performance deteriorated when the initial finger spacing did not match the object geometry. This limitation was most evident in the stable holding results. In contrast, the variable-stroke configuration produced consistently better performance across the tested objects. The improvement was not limited to one specific category. It appeared as a general increase in grasp reliability when the initial finger distribution was adjusted before bending.

For objects with relatively small grasping cross-sections, such as the orange and the Rubik’s cube, both configurations could achieve grasp initiation. The difference lay mainly in stability. When the initial stroke was reduced, the fingers formed a tighter enclosing posture, which improved contact uniformity and reduced the tendency of the object to shift during lifting. This effect was also observed for rectangular box A, where the benefit of variable-stroke adjustment was modest but still measurable in the stable holding results.

A more noticeable advantage appeared for cylindrical objects. In the fixed-stroke configuration, the beverage can and the water bottle were more sensitive to local slip and object tilting, especially when the initial contact occurred away from the preferred grasping region. After stroke adjustment, the fingers approached these objects from a more suitable initial geometry, which improved both grasp completion and retention. The representative images in Figure 8 also show that the hand could tolerate different contact configurations for cylindrical targets, indicating that the proposed mechanism improves not only size adaptability but also practical tolerance to pose variation.

The largest difference was observed for objects requiring a wider initial span. This trend is reflected most clearly in the results for the tissue pack, rectangular box B, and rectangular box C. Under the fixed-stroke configuration, these objects were more likely to experience insufficient enclosure or unstable lifting because the initial finger spacing was too limited. Once the base stroke was increased, the hand could establish contact from a more favorable initial arrangement, and the grasp became more stable. Although the largest rectangular target remained challenging, the variable-stroke configuration still yielded a clear improvement over the fixed-stroke case.

The result for Rectangular Box C requires further interpretation. Compared with the fixed-stroke configuration, the increased stroke improved the ability of the hand to reach and contact this larger object. However, the stable holding rate remained limited. This indicates that a larger geometric span improves accessibility, but it is not sufficient to guarantee stable retention. Rectangular Box C has a large and relatively flat grasping cross-section, which makes it difficult for the soft fingers to form deep enclosure around the object. In some trials, the fingers contacted the outer surfaces but did not generate sufficiently balanced multi-point contact during lifting. As a result, the object remained sensitive to local slip and contact imbalance.

Taken together, the results indicate that the advantage of the proposed design does not come from a stronger finger alone. The main gain arises from the ability to adapt the global finger layout to the object before grasping. This explains why the improvement becomes more evident as the mismatch between object size and initial finger spacing increases. The quantitative comparison in Table 3 therefore supports the central claim of this study, namely, that synchronous base-stroke adjustment enhances grasping adaptability at the system level.

### 5.3. Discussion on Workspace Enhancement and Grasp Adaptability

The results should be interpreted from the perspective of function separation between the rigid base and the soft fingers. Previous soft gripper studies have shown that compliant fingers can improve safe interaction and conformal contact with fragile or irregular objects [21]. However, when the finger layout is fixed, the initial grasping geometry remains constrained by the preset root positions of the fingers [22]. In that case, the fingers must rely mainly on bending deformation to compensate for object-size mismatch. The present design addresses this limitation by adding a pre-contact geometric reconfiguration stage. The rigid base first adjusts the radial distribution of the finger roots, and the soft fingers then provide local compliance during enclosure.

The overall comparison in Table 4 shows that the variable-stroke configuration outperformed the fixed-stroke configuration in both stable grasping and covered size range. This result is consistent with the workspace analysis in Section 3. The key point is that the proposed hand does not depend on finger bending alone to adapt to object size. Instead, stroke adjustment changes the initial distribution of the finger roots before contact occurs, and the subsequent bending motion then completes the local enclosure around the object.

This distinction is important. In a fixed-stroke configuration, the initial finger spacing remains unchanged for all targets. Once the object size deviates from that preset geometry, the grasping process becomes more sensitive to incomplete enclosure, local slip, and unstable lifting. This limitation becomes more evident for large targets and for objects whose grasping cross-sections differ substantially from the nominal hand geometry. The results in Table 3 reflect this trend. Several objects could still be contacted under the fixed-stroke condition, but stable retention was more difficult to maintain when the initial spacing was not suitable.

The variable-stroke configuration addresses this limitation at the system level. By adjusting the base stroke in advance, the hand approaches the object from a more suitable initial arrangement. This reduces the mismatch between finger spacing and object size before bending begins. As a result, the fingers do not need to compensate for the full geometric difference through deformation alone. The improvement observed in the experiments therefore comes from a better distribution of the finger roots rather than from any change in the finger structure itself.

The relationship between workspace enlargement and grasp performance should therefore be interpreted carefully. The proposed variable-stroke mechanism mainly improves geometric accessibility by changing the initial distribution of the finger roots. This allows the hand to approach objects with different grasping cross-sections from a more suitable configuration. However, geometric accessibility is only one factor affecting grasp performance. Stable holding further requires sufficient enclosure, balanced contact distribution, adequate normal force, and enough friction at the contact interface. Therefore, an enlarged workspace should not be interpreted as a direct guarantee of higher payload capacity or grasp stability.

This distinction explains the result for Rectangular Box C. Increasing the stroke expanded the initial grasping span and improved the grasp success rate, showing that the object became more reachable under the variable-stroke configuration. Nevertheless, stable holding remained difficult because the object presented a large, flat, and box-shaped cross-section. Under this condition, the fingers could contact the object but could not always wrap around it sufficiently to form a stable enclosing grasp. The remaining limitation is therefore mainly related to insufficient enclosure and unfavorable contact geometry. Force and friction constraints may also contribute during lifting, but they were not quantified in the present study because direct contact-force and payload-capacity measurements were beyond the scope of the current experiments.

The effect is most visible for targets that require a larger grasping span. For such objects, increasing the stroke expands the global grasping envelope and improves the likelihood of forming a stable enclosing contact. For smaller targets, reducing the stroke allows the hand to adopt a more compact configuration, which helps limit object displacement during lifting. In both cases, the underlying mechanism is the same: the base reconfiguration modifies the global geometry of the hand, while the soft fingers provide local compliance at the contact interface.

The present results therefore support the main claim of this study. The proposed mechanism improves grasping adaptability by introducing geometric reconfiguration at the hand level. This is the main difference between the present design and conventional fixed-base soft hands using the same type of fingers. The contribution of the variable-stroke mechanism is not simply a wider motion range in isolation, but a more appropriate initial grasping geometry across objects with different grasping cross-sections.

Compared with compliance-based soft grippers, the proposed design does not rely only on passive deformation after contact. Instead, it modifies the initial hand geometry before contact is established. Compared with underactuated adaptive hands, the present mechanism does not introduce additional finger joints or tendon-coupled phalanges; the soft fingers remain structurally unchanged, and the reconfiguration occurs at the base level. Compared with rigid-body reconfigurable hands, the proposed mechanism uses a shared synchronous transmission rather than independent finger-positioning modules. This reduces mechanical and control complexity, while still allowing the hand to tune its grasping span for objects with different cross-sectional sizes. Therefore, the main contribution is not simply that the hand has a larger workspace, but that the workspace can be mechanically reconfigured through a compact, coupled, and hand-level transmission mechanism.

The current study is limited to static grasping under open-loop pneumatic actuation. The reported results verify the structural and functional validity of the proposed concept, but they do not yet provide direct measurements of contact force, friction margin, payload capacity, long-term reliability, or dynamic manipulation performance. These aspects will be considered in future work through force-sensing integration, payload-capacity testing, closed-loop pneumatic control, and repeated grasping experiments under different surface conditions.

## 6. Conclusions

This study presented a four-finger pneumatic soft robotic hand with a synchronous variable-stroke base mechanism for improving grasping adaptability through hand-level geometric reconfiguration. A single swing-cylinder input drives four finger roots synchronously over an 80 mm radial stroke via a shaft-flange and arc-link transmission, without modifying the soft-finger structure. A system-level kinematic model integrating rigid-base reconfiguration with constant-curvature finger bending was established and experimentally verified, with span errors below 1.4% across all tested configurations. Comparative grasping experiments demonstrated that the variable-stroke configuration raised the mean grasp success rate from 62.5% to 95.0% and expanded the stably graspable size range from 2.0 cm to 6.0 cm relative to the fixed-stroke baseline. These results confirm that matching the initial finger spacing to the target object geometry, rather than relying on finger deformation alone, is the primary source of performance gain. The proposed design is therefore most suitable for moderate-payload grasping tasks involving objects with variable sizes, irregular geometries, or fragile surfaces. The current prototype is limited to pneumatic soft bending fingers, static laboratory grasping, and manually preset stroke adjustment. Future work will focus on programmable stroke selection, actuator-interface compatibility, contact-force and payload characterization, long-term durability and failure-rate evaluation, and grasping experiments under more demanding surface and environmental conditions.

## Figures and Tables

**Table 1:** Masses and dimensions of the tested objects used in the grasping experiments.

No.	Item	Weights (g)	Dimensions (cm)
1	Orange	192	Diameter: 11
2	Rectangular Box A	201	6 × 4 × 4
3	Beverage Can	165	16 × 14 × 10
4	Water Bottle	23	21 × 6 × 6
5	Rubik’s Cube	64	5 × 5 × 5
6	Tissue Pack	168	13 × 6 × 10
7	Rectangular Box B	156	20 × 20 × 6
8	Rectangular Box C	174	23 × 1 × 6

**Table 2:** Theoretical and measured geometric spans under different stroke configurations.

Stroke Configuration	Stroke s (mm)	Root Radius ρ(s) (mm)	$\mathrm{Theoretical}\;\mathrm{Opposite}\;\mathrm{Span}\;D_{opp}$(mm)	Measured Opposite Span (mm)	$\mathrm{Error}\;\mathrm{in}\;D_{opp}$ (%)	$\mathrm{Theoretical}\;\mathrm{Adjacent}\;\mathrm{Span}\;D_{adj}$ (mm)	Measured Adjacent Span (mm)	$\mathrm{Error}\;\mathrm{in}\;D_{adj}$ (%)
Nearest-stroke	0	65	130.00	129.4	0.46	91.92	93.0	1.18
Middle-stroke	40	105	210.00	208.8	0.57	148.49	146.9	1.07
Farthest-stroke	80	145	290.00	287.6	0.83	205.06	202.3	1.35

**Table 3:** Grasping performance of the proposed hand under fixed and variable stroke settings ($L_{c}$, characteristic size in the grasping cross-section; $s_{f}$, fixed stroke; $s_{v}$, variable stroke; n, number of trials; $N_{g}$, successful grasps; $N_{s}$, stable grasps; $\eta_{g}$, grasp success rate; $\eta_{s}$, stable holding rate).

Object	$L_{c}$	Mass	$s_{f}$	$s_{v}$	n	$N_{g,f}$	$\eta_{g,f}$	$N_{s,f}$	$\eta_{s,f}$	$N_{g,v}$	$\eta_{g,v}$	$N_{s,v}$	$\eta_{s,v}$	Observation
Orange	11.0	192	40	20	5	2	40	0	0	5	100	4	80	Better after stroke reduction
Rectangular box A	6.0	201	40	15	5	5	100	4	80	5	100	5	100	More uniform contact
Beverage can	7.0	165	40	25	5	4	80	3	60	5	100	4	80	Better posture tolerance
Water bottle	6.0	23	40	25	5	3	60	2	40	5	100	4	80	Reduced slip
Rubik’s cube	5.0	64	40	10	5	5	100	4	80	5	100	5	100	Stable in both modes
Tissue pack	10.0	168	40	45	5	3	60	2	40	5	100	4	80	Better retention
Rectangular box B	10.0	156	40	55	5	2	40	1	20	4	80	3	60	Improved by span increase
Rectangular box C	12.0	174	40	65	5	1	20	0	0	4	80	2	40	Still challenging

**Table 4:** Overall comparison between fixed-stroke and variable-stroke configurations.

Configuration	Number of Tested Objects	Number of Successfully Grasped Objects	Number of Stably Held Objects	Minimum Stable Characteristic Size (cm)	Maximum Stable Characteristic Size (cm)	Covered Size Range (cm)	Mean Success Rate (%)	Mean Stable Holding Rate (%)	Main Limitation
Fixed-stroke	8	5	3	5.0	7.0	2.0	62.5	40.0	Limited initial span for large objects; unstable retention for deformable and cylindrical targets
Variable-stroke	8	8	7	5.0	11.0	6.0	95.0	77.5	Performance still decreases for the largest box-shaped target

## Data Availability

The authors do not have permission to share data.
